# Commentary: Targeting fibroblast activation protein in rheumatoid arthritis: from molecular imaging to precision therapeutics

**DOI:** 10.3389/fimmu.2025.1653274

**Published:** 2025-12-16

**Authors:** Yaoxin Ao, Jiangfeng Lyu, Junxing Yang, Fangjun Xiao

**Affiliations:** Shenzhen Hospital (Futian) of Guangzhou University of Chinese Medicine, Shenzhen, Guangdong, China

**Keywords:** artificial intelligence, diagnostic specificity, dual-tracer pet, FAP-targeted PET/CT, rheumatoid arthritis

## Introduction

The precision diagnosis and treatment of rheumatoid arthritis (RA) urgently requires breakthroughs beyond the limitations of conventional imaging techniques. A recent review by Huang et al., published in Frontiers in Immunology, systematically summarizes significant advances in fibroblast activation protein (FAP)-targeted molecular imaging in RA. The authors highlight the high sensitivity of FAP inhibitor (FAPI) PET/CT in detecting synovitis and its promising translational value for disease assessment and therapeutic monitoring. The review underscores that FAP is specifically overexpressed in activated synovial fibroblasts, the key pathogenic cells in RA. Therefore, FAPI PET/CT enables noninvasive visualization of joint inflammation and may offer a new diagnostic approach for seronegative RA. However, the discussion remains limited regarding the diagnostic specificity challenges that FAPI imaging may encounter in real-world clinical practice.

## General comments

Recent clinical evidence suggests that FAP is also markedly expressed in various non-RA pathological conditions, raising concerns about low specificity despite high sensitivity and increasing the likelihood of widespread false positives. For instance, Fenercioğlu et al. reported a direct comparison in a patient with metastatic melanoma and concomitant knee osteoarthritis (OA), where ^68^Ga-FAPI-4 uptake in the joint markedly exceeded that of ^18^F-FDG (1). Similarly, FAPI uptake has been observed in granulomatous diseases (e.g., hepatic sarcoidosis reported by Araz) and benign ossification processes (e.g., gluteal myositis ossificans reported by Al-Rashdan) (2, 3). A review by Bentestuen et al. further noted that (4) among 2,372 non-malignant FAPI-positive lesions, musculoskeletal and joint-related abnormalities accounted for 10%—second only to atherosclerosis (49%)—encompassing inflammation, tuberculosis, periodontitis, and healing wounds. These findings highlight the broad biological basis of FAPI and its resulting diagnostic dilemmas. This challenge is particularly pronounced in differentiating RA from OA. A prospective study by Mu et al. showed that (5) the median SUVmax of ^18^F-FAPI uptake in active RA joints was 3.6, significantly higher than that of healthy controls (2.4). However, Yang et al. reported that (6) ^68^Ga-FAPI uptake in OA-affected thoracic facet joints could also reach a SUVmax of 3.6, indicating a potential risk of significant value overlap between the two conditions. It is noteworthy that studies indicate a generally higher synovial FAP expression in RA than in OA. Bauer et al. reported significantly elevated FAP mRNA and protein in RA synovium compared to OA, localized to myofibroblast-like synoviocytes co-expressing key degradative enzymes (7). Similarly, Wäldele et al. observed abundant FAP expression throughout RA synovial membranes, contrasting with minimal expression in OA samples (8). While these findings suggest a differential expression profile, the studies are limited by sample size and potential sampling bias from end-stage disease tissues. Consequently, future efforts should prioritize large-scale, head-to-head comparisons in well-characterized cohorts to determine if a validated diagnostic cutoff for FAPI uptake (e.g., SUVmax) can be established to reliably distinguish RA from OA.

Pending the establishment of such a diagnostic cutoff, the low specificity of FAPI PET/CT remains a clinical challenge. It can misinterpret benign signals (e.g., from OA) as RA, leading to misdiagnosis and unnecessary treatment. Even after RA confirmation, these background signals can interfere with inflammation assessment and treatment evaluation, making it difficult to distinguish new RA lesions from other conditions.

To improve diagnostic specificity, dual-tracer PET/CT strategies have demonstrated substantial potential. Wegen et al. found that combining ^68^Ga-FAPI-46 with ^18^F-FDG in cancer patients improved lesion-to-background ratios and functional tumor volume (9). Liu et al. proposed a “one-stop” low-dose dual-tracer protocol (^18^F-FDG 0.37 MBq/kg + ^68^Ga-DOTA-FAPI-04 0.925 MBq/kg), which significantly enhanced metastasis detection rates while maintaining acceptable radiation exposure (10). Zheng et al. further validated the feasibility of a 34-minute rapid scan protocol (11). Notably, optimization of probe targets has also yielded promising results. Wang et al. developed a dual-target probe (12), ^18^F-AlF-FAPI-RGD, which binds both FAP and integrin αvβ_3_. By focusing on RA-specific microenvironmental features such as neoangiogenesis, this probe achieved a joint detection rate of 82.4%, significantly outperforming physical examination and effectively avoiding OA-related interference.

The rise of artificial intelligence (AI) offers new opportunities for advancing imaging analysis. Yan et al. developed a deep learning radiomics model integrating ultrasound and radiomic features, achieving an AUC of 0.979 in detecting RA-related bone erosion, demonstrating the potential of multi-feature synergy in differential diagnosis (13). If adapted to FAPI imaging, this approach could be further enhanced by integrating joint 3D morphological data from high-resolution peripheral quantitative CT (HR-pQCT). Folle et al. (14) demonstrated the ability of deep learning models using joint bone shapes (AUROC for RA: 75.4%) to differentiate arthritis types, highlighting the potential of combining this structural data with FAPI’s molecular information in a bimodal model to mitigate FAP’s limited diagnostic specificity. There is both theoretical and practical foundation for developing a radiomics model based on FAPI imaging: FAPI offers a RA-specific molecular target, radiomics enables quantitative lesion characterization, and AI algorithms enhance classification performance. However, this approach currently faces challenges related to standardization. Ebrahimpour et al. demonstrated significant discrepancies in feature extraction between radiomics libraries (e.g., PyRadiomics vs. RaCat), with only 75 of 1,665 features being shared, and further showed that feature-direction relationships with clinical endpoints were affected by grayscale discretization parameters (15). Demircioğlu also pointed out that the high dimensionality and small sample size in current studies may limit model reproducibility and hinder clinical translation (16). Therefore, multicenter prospective studies are needed to validate the efficacy and translational value of FAPI-based imaging in distinguishing RA from other joint disorders.

## Discussion

In summary, although Huang et al. have comprehensively reviewed major advances in the field, addressing specificity concerns is essential for the clinical application of FAPI PET/CT. We encourage the authors to further elaborate on these aspects in future updates to support a more robust framework for precision medicine in RA.
